# Identity Disturbance in Borderline Personality Disorder: A Scoping Review Protocol

**DOI:** 10.31083/AP39864

**Published:** 2025-04-21

**Authors:** Danielle Mullins, Sonia Lorente, Víctor Suárez, Guillem Feixas

**Affiliations:** ^1^Department of Clinical Psychology and Psychobiology, University of Barcelona, 08035 Barcelona, Spain; ^2^Faculty of Psychology, Autonomous University of Barcelona, 08193 Barcelona, Spain; ^3^Faculty of Health Sciences, Isabel I University, 09003 Burgos, Spain; ^4^Department of Psychiatry, Vall d’Hebron University Hospital, 08035 Barcelona, Spain; ^5^Institute of Neurosciences, University of Barcelona, 08035 Barcelona, Spain

**Keywords:** conceptualization, self-identity, psychopathology, emotional instability

## Abstract

**Background:**

Borderline personality disorder (BPD) is a complex mental health condition characterized by instability in interpersonal relationships, affect regulation, and impulse control. A core feature of BPD is identity disturbance, marked by a persistently unstable self-image and sense of self. Despite clinical recognition, the precise nature of identity disturbance remains ambiguous, with no clear consensus on its specific manifestations and measurable parameters. With the scoping review projected in this protocol, we aim to define what has been said in the literature about identity disturbance, its types, and methods of measurement.

**Study Design:**

Scoping review protocol.

**Methods:**

Included publications will comprise empirical and theoretical studies focusing on identity disturbance in individuals diagnosed with BPD. Databases will include PubMed, PsycINFO, Scopus, and Web of Science Core Collection, supplemented by Google for grey literature. Data will be screened by two reviewers and discrepancies will be resolved through discussion or a third reviewer if necessary. Extracted data will include study details, methodological data, definitions of identity, conceptualizations of identity disturbance, types of disturbance, and assessment tools.

**Conclusions:**

The forthcoming results will have the potential to make significant contributions to both theoretical and empirical knowledge on identity disturbance in BPD. It is expected that the results of the review will help to inform and improve therapeutic strategies, enabling more tailored and effective interventions for BPD patients with identity disturbance.

## Main Points

• This review will address a critical gap in understanding the specific manifestations and measurable parameters of identity disturbance, a core feature of borderline personality disorder (BPD).

• This review will identify the definitions, types, and measurement approaches for identity disturbance in individuals with BPD, offering a detailed map of the current knowledge that can inform clinical practice and future research.

• The findings are expected to have significant implications for research and practice, including a clarification of the concepts involved in identity disturbance in people with BPD, and a compendium of the measurement approaches used.

## 1. Introduction

Borderline personality disorder (BPD) is a complex mental health condition that is characterized by instability in a number of areas including interpersonal relationships, affect regulation, impulse control, and sense of identity [1, 2]. Precisely, identity disturbance, which involves a markedly and persistently unstable self-image or sense of self, is one of the core features of BPD [1]. It is well established in the literature and clinical practice that individuals with BPD experience identity issues [3, 4, 5]. Identity disturbance is manifested in difficulty experiencing agency, unreflective movement from one present moment to another, a profound sense of inauthenticity regarding self-concept, and feeling disconnected from others and excluded from social communities [6]. It has been previously demonstrated that identity disturbance in BPD patients is an important indicator of symptom severity [7] and interpersonal problems [8], as well as being correlated with emotion dysregulation [9].

However, the nature of identity has not been clearly or precisely conceptualized, as evidenced by the DSM-5’s vague description of the term [1]. While identity is commonly understood as a continuing sense of self [10], the ambiguity of this definition complicates the task to define and study its disturbance [11]. It has been recognized that identity disturbance is a multifaceted construct associated with a variety of maladaptive outcomes, however, its underlying mechanisms and potential interventions remain under investigated. Furthermore, there remains a lack of agreement regarding the specific manifestations and measurable parameters of identity disturbance [12]. For example, some authors argue that the lack of stability in identity is a manifestation of identity conflicts in the meaning system of these patients (e.g., implicit dilemmas) [13], which can be measured and identified, while others view instability as an “intrinsic” characteristic of BPD to be considered only in terms of its behavioral consequences.

Given the broad range of literature and the multifaceted nature of this concept [14], a scoping review is particularly suitable for shedding light on this topic. Scoping reviews are designed to map the breadth and depth of existing literature, clarify key concepts and definitions, and identify knowledge gaps on a given topic [15, 16]. This approach will allow for a comprehensive examination of the available knowledge of identity disturbance in BPD, including diverse theoretical perspectives, study designs, methodologies, and main results.

Identifying the various meanings and types of identity disturbance in the current literature may provide further clarification of this concept. Several terms have been used interchangeably or in connection to identity disturbance, including identity diffusion, disturbed sense of self, and identity pathology. Some researchers have also defined identity as narrative, and in BPD, identity disturbance manifests through incoherent self-narratives that are both a symptom and a constitutive part of the disorder. In reading several articles on this topic, we found that an array of different types of identity disturbance were described. For example, Marcia [17] has described identity statuses (i.e., achievement, foreclosure, moratorium, diffusion) that demonstrate the continuum of identity development and the factors that may be central components to identity disturbance. Additionally, Wilkinson-Ryan and Westen [18] found four different aspects of identity disturbance that impact adults with BPD—role absorption, painful incoherence, inconsistency, and lack of commitment.

Moreover, we expect to identify assessment tools that measure identity disturbance that may help define or provide insight into the concept and produce empirical evidence for its role in BPD. After a brief review of the current literature, we found that identity disturbance in BPD is often assessed by extracting individual items from BPD scales (e.g., Diagnostic Interview for DSM-IV Personality Disorders). With this scoping review, we plan to create a comprehensive list of the current assessment tools available, and to provide information on which are most frequently used and the reliability of the instruments.

Our potential contribution should help advance the knowledge of identity disturbance in patients diagnosed with BPD. This has the potential to improve psychotherapeutic outcomes in this population, as a stable and consolidated identity has been recognized as an essential factor for psychological resilience, self-regulation, and the ability to navigate one’s social world [6, 19]. Considering that we will gather evidence from different theoretical backgrounds, the forthcoming findings will allow us to offer a heuristic summary, a comprehensive view of this concept.

## 2. Method

**Fig. 1.**
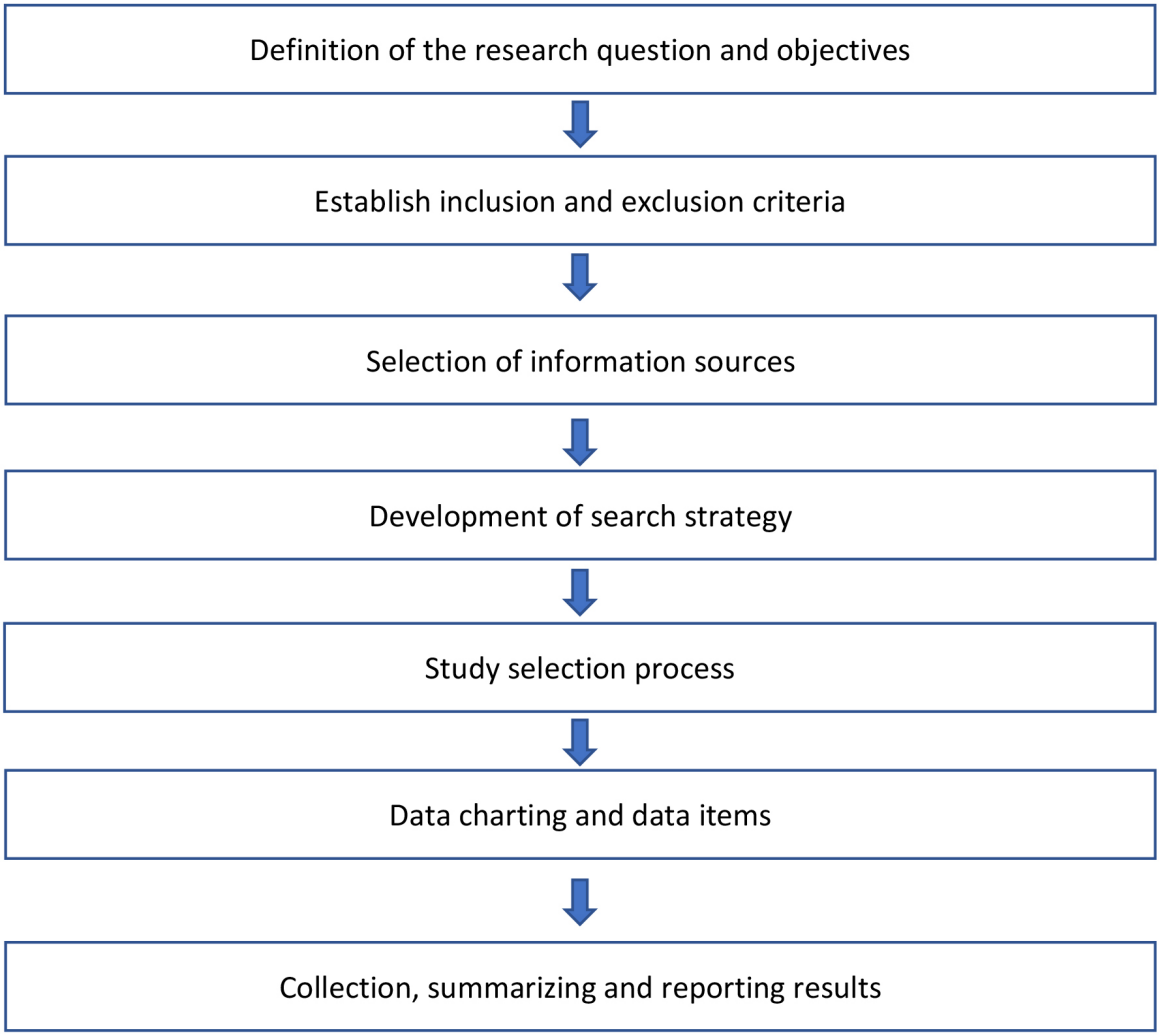
**Flowchart of the review process**.

The methodological framework proposed by Arksey and O’Malley [20], updated by Levac and colleagues [21], will be used to guide the scoping review. The Preferred Reporting Items for Systematic Reviews and Meta-Analysis for protocols (PRISMA-P) [22] and the extension for scoping reviews (PRISMA-ScR) [23] will be used to ensure the quality of reporting. The prior version of this protocol was registered in the Open Society Framework (OSF), available at https://doi.org/10.17605/OSF.IO/6WYQT. Fig. 1 shows the whole process of the review.

### 2.1 Research Question and Objectives

The research question seeks to explore what is discussed in the literature regarding identity disturbance, its types, and methods of measurement. Thus, the primary objective of this scoping review is to define what has been said in the literature about patients diagnosed with BPD and identity disturbance (Population), types of identity disturbance, including its specific manifestations, categories, and descriptions that distinguish it from previous definitions (Concept), and methods used for its measurement (Context). This objective was defined according to the PCC mnemonic (Population, Concept and Context) as recommended for scoping reviews [16].

The specific objectives that this review aims to achieve are:

(1) To clarify how authors define identity disturbance in patients with BPD.

(2) To identify what other concepts or terms have been used to describe identity disturbance in this population.

(3) To determine types of identity disturbance described in the literature for BPD patients.

(4) To Identify the instruments used to assess identity disturbance.

### 2.2 Eligibility Criteria

We will search for both empirical and theoretical studies that address the relationship between BPD and identity disturbance, and that focus on identity disturbance itself (e.g., defining, discussing types, and/or measuring it). Concerning the empirical studies, both experimental and quasi-experimental study designs will be considered, including randomized controlled trials, non-randomized controlled trials, cross-sectional and cohort studies. We also will consider studies focused on qualitative data. The present review will include publications in which the participants are individuals diagnosed with BPD by a qualified professional using established diagnostic criteria and/or clinical assessment tools. We will include studies written in English, Spanish, Catalan, Italian, and Portuguese. We will exclude publications that do not provide a definition of identity disturbance, talk about types or provide a measure of them. Systematic reviews, meta-analyses, reports, conferences, letters to the editor and opinions will also be excluded.

### 2.3 Information Sources 

The scoping review will be conducted through subject-specific and multidisciplinary databases as outlined. PubMed is a subject-specific database that dedicated to medicine and related sciences (US National Library of Medicine, National Center for Biotechnology Information). This database includes high-quality qualitative and quantitative research to ensure a well-rounded approach to understanding identity disturbance in BPD from a medical perspective. Likewise, PsycINFO (ProQuest) is a subject-specific database that encompasses a wide range of psychological literature, including studies that explore psychological theories, clinical practices, cognitive and emotional processes, and the social context of identity disturbance. By using PsycINFO, the review can integrate diverse perspectives that will allow for an in-depth examination of the intricacies of identity disturbance in BPD and their implications for treatment. The multidisciplinary databases Scopus (Elsevier) and the Web of Science Core Collection (Clarivate) will be included as they offer a vast array of literature across various fields, including psychology, social sciences, and medicine. The rigorous indexing from these databases will ensure that credible and relevant research is included. This capacity is essential for a comprehensive exploration of the scholarly discourse surrounding identity disturbance in BPD. In addition, Google (up to 100 results) will be used to search grey literature. By incorporating these databases, the scoping review will have the capacity to create a well-rounded, evidence-based understanding of identity disturbance in BPD. If needed, authors of eligible studies will be contacted to provide missing or additional data.

### 2.4 Search Strategy

The search strategy, developed in accordance with the stated objectives and inclusion criteria, is presented in Appendix Table 1. The search terms have been adapted for every database. No restrictions on publication years will be set.

### 2.5 Study Selection

References identified by the search strategy will be entered into Rayyan bibliographic software and duplicates will be removed. Titles and abstracts, and study selection, will be screened independently by two reviewers. If decisions cannot be made from the title and abstract alone, the full paper will be retrieved. Agreement between reviewers in both phases will be analyzed using Cohen’s kappa [24]. Disagreements during the process will be resolved by discussion (with a third reviewer if necessary).

### 2.6 Data Charting and Data Items

As shown in Appendix Table 2, the Microsoft Excel template has been designed for data extraction, where the key study characteristics and findings will be documented. The structured format will feature different sections for recording information, including one for selecting studies based on the inclusion criteria and another for extracting relevant data. Regular backups and version control will be maintained to safeguard data and track changes. The following data items will be extracted from the publications:

(a) General information (author, title, year, country).

(b) Methodological data (type of design, aim of the study, sample size).

(c) Sample characteristics such as age, sex, gender, diagnosis.

(d) Identity conceptualization (definition of identity, if provided).

(e) Background (theoretical framework supporting their definition of identity disturbance, if provided).

(f) Identity disturbance conceptualization (definition(s) of identity disturbance, if provided).

(g) Types of identity disturbance (types and their definitions, if considered).

(h) Assessment of identity disturbance (name of measurement tool and description of what it measures exactly, validity and reliability).

In order to manage the different languages, the review will leverage the language proficiencies of the research team. This team includes one native English-speaking co-author, with the other co-authors possessing full professional competence in the language, who will assist in the data extraction for English studies. For non-English studies, the team demonstrates native and/or full professional competence in the respective languages (Spanish, Catalan, Italian, and Portuguese). To ensure consistency across languages, team meetings will be held to resolve any translation ambiguities or inconsistencies. When necessary, translations will be cross-checked by team members to confirm accuracy and maintain uniformity in the data extraction.

### 2.7 Risk of Bias in Individual Studies

The risk of bias in individual studies and the quality of evidence in scoping reviews is not mandatory and will not be conducted.

### 2.8 Ethics and Dissemination

This scoping review protocol does not require approval from an ethics committee, as it does not involve human experimentation. The ethical implications of the results may be related to potential significant contributions to both theory and research on identity disturbance in BPD. Dissemination of results will consist of presentations at national and international conferences in the fields of psychology and psychiatry. Similarly, the final report of findings will be submitted to international and peer-reviewed journals.

### 2.9 Timeline

The study selection stage, including title and abstract screening and full-text selection is expected to take four months. The data charting stage is expected to take about five months, and the summarizing and reporting of results is expected to take three months. An update of the search will be conducted prior to the submission of the final report, which is anticipated to be available one year after the commencement of the entire process.

### 2.10 Collating, Summarizing and Reporting Results

A PRISMA flow diagram will be included to report each stage of the review process [23]. Narrative information will be categorized in key sections, including general information, methodological data, and specific data relating to the scoping review objectives in order to provide an exhaustive report of the conceptualizations of identity disturbance and its types. We will also report the methods in which identity disturbance can be measured. The data will be presented in figures and tables, as appropriate. This work will not involve interpretation of the data extracted from any of the perspectives used, nor will it involve statistical analysis of the various results reported in the studies.

## 3. Conclusions

The forthcoming results of this scoping review protocol have the potential to make significant contributions to both theory and research concerning identity disturbance in BPD. By mapping the manifestations of identity disturbance in BPD patients, the review aims to provide clinicians and researchers with a more comprehensive understanding of the condition. Moreover, this deeper insight is crucial to develop effective interventions for patients [3]. It is expected that this review will help inform and enhance therapeutic strategies, enabling more tailored and effective interventions for BPD patients with identity disturbance. Clinicians will be better equipped to address the specific identity-related challenges faced by individuals with BPD, potentially leading to improved patient outcomes.

A critical aspect of this review is the identification of the tools currently used to measure identity disturbance in BPD. This evaluation will inform stakeholders of the quality of assessment tools to potentially minimize underestimation of diagnostic stability caused by measurement error [25]. Previous research has shown that it is crucial to have tools that accurately distinguish normal identity problems from clinical identity disturbance for early prevention and intervention purposes [26]. These advancements will not only help clarify the most appropriate assessment tools for clinical practice, but they will support more robust and insightful future research.

From an investigative perspective, the review will map the existing knowledge and definitions of identity disturbance, as well as highlighting inconsistencies and areas that require further exploration. This comprehensive mapping will help clarify and standardize the definition of identity disturbance in BPD, which is essential for advancing research and facilitating comparable study outcomes. Furthermore, this review will guide future research by identifying underexplored areas, encouraging further investigation into aspects that remain unclear. Overall, the anticipated findings from this scoping review have far-reaching implications for both clinical practitioners and research. With this review, we aim to enhance the understanding of identity disturbance in BPD, from a clarified conceptual framework to practical treatment recommendations. These developments will contribute to improved patient care and more informed investigative efforts.

## Availability of Data and Materials

Data sharing not applicable — no new data generated since this is a protocol.

## Supplementary Material

Supplementary material associated with this article can be found, in the online version, at https://doi.org/10.31083/AP39864.

## Appendix

See Tables 1,2.

**Appendix Table 1.** **Search strategy**.

Database	Search script
Scopus	TITLE-ABS-KEY (BPD OR “Borderline Personality Disorder” OR EUPD OR “emotionally unstable personality disorder”) AND TITLE-ABS-KEY (“identity disturbance*”)
WOS	TS (BPD OR “Borderline Personality Disorder” OR EUPD OR “emotionally unstable personality disorder”) AND TS= (“identity disturbance*”)
PubMed	ALL FIELDS (BPD OR “Borderline Personality Disorder” OR EUPD OR “emotionally unstable personality disorder”) AND (“identity disturbance”)
PsycINFO	ANY FIELDS (BPD OR “Borderline Personality Disorder” OR EUPD OR “emotionally unstable personality disorder”) AND (“identity disturbance*”)
Google	(BPD OR “Borderline Personality Disorder” OR EUPD OR “emotionally unstable personality disorder”) AND (“identity disturbance*”) pdf

*Note*. Scopus, by Elsevier; WOS = Web of Science CORE, by Clarivate; PubMed, US National Library of Medicine, by National Center for Biotechnology Information (NCBI); PsycINFO = Psychological Information, by ProQuest. No restrictions on publication years will be set.

**Appendix Table 2.** **Data extraction template**.

Source	INCLUDED (Y/N)	Objective	Design	Sample (sex, gender, age)	Diagnoses	Identity (definition)	Identity disturbance (definition)	Types of identity disturbance	Instruments (Validity and Reliability)	Conclusions

*Note.* Columns (e.g., instruments) are replicable as many times as necessary.
